# Genome-Wide Analysis of the Soybean TIFY Family and Identification of *GmTIFY10e* and *GmTIFY10g* Response to Salt Stress

**DOI:** 10.3389/fpls.2022.845314

**Published:** 2022-03-23

**Authors:** Ya-Li Liu, Lei Zheng, Long-Guo Jin, Yuan-Xia Liu, Ya-Nan Kong, Yi-Xuan Wang, Tai-Fei Yu, Jun Chen, Yong-Bin Zhou, Ming Chen, Feng-Zhi Wang, You-Zhi Ma, Zhao-Shi Xu, Jin-Hao Lan

**Affiliations:** ^1^College of Agronomy, Qingdao Agricultural University, Qingdao, China; ^2^Institute of Crop Science, Chinese Academy of Agricultural Sciences (CAAS)/National Key Facility for Crop Gene Resources and Genetic Improvement, Key Laboratory of Biology and Genetic Improvement of Triticeae Crops, Ministry of Agriculture, Beijing, China; ^3^Hebei Key Laboratory of Crop Salt-Alkali Stress Tolerance Evaluation and Genetic Improvement/Cangzhou Academy of Agriculture and Forestry Sciences, Cangzhou, China

**Keywords:** soybean, TIFY, salt tolerance, ABA, transcription factor

## Abstract

TIFY proteins play crucial roles in plant abiotic and biotic stress responses. Our transcriptome data revealed several *TIFY* family genes with significantly upregulated expression under drought, salt, and ABA treatments. However, the functions of the *GmTIFY* family genes are still unknown in abiotic stresses. We identified 38 *GmTIFY* genes and found that *TIFY10* homologous genes have the most duplication events, higher selection pressure, and more obvious response to abiotic stresses compared with other homologous genes. Expression pattern analysis showed that *GmTIFY10e* and *GmTIFY10g* genes were significantly induced by salt stress. Under salt stress, *GmTIFY10e* and *GmTIFY10g* transgenic *Arabidopsis* plants showed higher root lengths and fresh weights and had significantly better growth than the wild type (WT). In addition, overexpression of *GmTIFY10e* and *GmTIFY10g* genes in soybean improved salt tolerance by increasing the PRO, POD, and CAT contents and decreasing the MDA content; on the contrary, RNA interference plants showed sensitivity to salt stress. Overexpression of *GmTIFY10e* and *GmTIFY10g* in *Arabidopsis* and soybean could improve the salt tolerance of plants, while the RNAi of *GmTIFY10e* and *GmTIFY10g* significantly increased sensitivity to salt stress in soybean. Further analysis demonstrated that *GmTIFY10e* and *GmTIFY10g* genes changed the expression levels of genes related to the ABA signal pathway, including *GmSnRK2*, *GmPP2C*, *GmMYC2*, *GmCAT1*, and *GmPOD*. This study provides a basis for comprehensive analysis of the role of soybean *TIFY* genes in stress response in the future.

## Introduction

Environmental stresses affect both growth and yield in soybean (Bohnert et al., 1995). To adapt to environmental stresses, several regulatory pathways gradually formed during the evolution of plants (Zhu et al., 2011). In previous studies, TIFY proteins were found to respond to abiotic and biotic stresses through regulatory pathways (Thines et al., 2007; Ebel et al., 2018). Studying TIFY proteins were useful for protecting soybean (*Glycine max*) growth and yield under various environmental stresses.

TIFY proteins were defined with conservative amino acid (aa) sequence (TIF[F/Y] XG) (Vanholme et al., 2007). The TIFY family genes were divided into four subfamilies, including TIFY, Jasmonate ZIM domain (JAZ), PEAPOD (PPD), and ZIM-like (ZML) according to their specific domains (Bai et al., 2011). TIFY subfamily members contain only one TIFY domain; JAZ subfamily members have a C-terminal Jas (SLX_2_FX_2_KRX_2_RX_5_PY) domain (also named CCT_2 domain) in addition to the TIFY domain (Staswick, 2008); ZML subfamily members contain the CCT domain (CONSTANS, CO-like, and TOC1) and the GATA zinc finger domain (CX_2_CX_20_CX_2_C), except for the TIFY domain (Nishii et al., 2000); PPD subfamily members contain the N-terminal PPD domain, TIFY domain and the C-terminal Jas domain without PY motif (SLX_2_FX_2_KRX_2_RX_5_) (Chung et al., 2009).

In the early research, TIFY proteins could respond to biotic stresses, such as insects and pathogens by jasmonic acid (JA) signaling pathway (Thines et al., 2007; Barah and Bones, 2015; Thireault et al., 2015; Mao et al., 2017; Dhakarey et al., 2018). Recent studies have demonstrated that TIFY proteins play an important role in regulating plants resistance to abiotic stresses (Demianski et al., 2012; Zhu et al., 2013; Fu et al., 2017; Sun et al., 2017; Peethambaran et al., 2018; Meng et al., 2019; Luo et al., 2020; Zhao et al., 2020a). The *Arabidopsis AtTIFY10a* and *AtTIFY10b* genes and their wild soybean homologous genes *GsTIFY10a, GsTIFY10b*, and *GsJAZ2* positively regulated the response to salt and alkali stresses (Zhu et al., 2013; Zhao et al., 2020a). Overexpression of *GhJAZ2* in cotton plants can significantly enhance sensitivity to salt stress (Sun et al., 2017). During the seedling and reproductive stages of rice, overexpression of *OsJAZ1* in rice can improve sensitivity to drought stress, while *JAZ1* t-DNA inserted in mutant plants had higher drought resistance than wild type (WT) plants (Fu et al., 2017). The rice *OsJAZ8* gene was confirmed to improve the salt tolerance of transgenic tobacco through the JA signaling pathway (Peethambaran et al., 2018). The hard wheat *TdTIFY11a* gene can improve salt tolerance when overexpressed in *Arabidopsis* (Ebel et al., 2018). *Arabidopsis AtJAZ7* gene was identified to mediate drought tolerance through comparative proteomics and metabolomics analysis (Meng et al., 2019). Cotton *GbJAZ1* gene was confirmed to interact with ABA-insensitive1 (*ABI1*) and involved in regulation the tolerance of salt and drought through the ABA signaling pathway (Luo et al., 2020).

Soybean is one of the most important commercial crops worldwide and an important source of vegetable protein and oil for humans. Salt stress is an important factor which could affect the growth and yield of soybean (Zhu et al., 2013). Studying salt stress-related genes and their functions are of great significance to soybean molecular breeding. After analyzing the transcriptome data in previous studies, we found that the expression levels of many *TIFY* family genes were significantly upregulated under drought, salt, and ABA treatments (Shi et al., 2018). In our study, we performed a genome-wide identification of the *TIFY* family genes in soybean and identified 38 *GmTIFY* genes. We analyzed the structure characteristics, expression patterns, duplication events, and physical and chemical properties of *GmTIFY* family genes. During transcriptome data analysis, we found six significantly upregulated genes under salt treatment, which were all *GmTIFY10* and *GmTIFY11* homologous genes in the JAZ subfamily. The gene function analysis of *GmTIFY10e* and *GmTIFY10g* showed that they have a positive regulatory effect on salt stress tolerance in *Arabidopsis* and soybean. Further analysis demonstrated that overexpression of *GmTIFY10e* and *GmTIFY10g* could influence the expression levels of ABA-related genes, which suggested that *GmTIFY10e* and *GmTIFY10g* may regulate the salt tolerance in plants by participating in ABA signaling pathway.

## Materials and Methods

### Screening and Identification of *TIFY* Genes

The nucleic acid and protein databases of *Arabidopsis*, rice, soybean, apple, and grape were downloaded from the Ensemble Plants database.^1^ The hidden Markov model (HMM) of TIFY domain (PF06200) was obtained from Pfam.^2^ We then used the hmm-search program HMMER3.1 (Prince and Pickett, 2002; Xia et al., 2017) to identify the TIFY HMM for the TIFY proteins in the resulting protein databases. The 18 *Arabidopsis* AtTIFY protein sequences were obtained from TAIR^3^ and used to search the TIFY proteins from rice, soybean, apple, and grape protein databases by the BLASTp program of basic local alignment search tool (BLAST; Wang et al., 2017). We compared the results of the two methods to confirm *TIFY* candidate genes in these species. These candidate genes were identified in their domains with SMART^4^ and CDD^5^ to ensure that the TIFY domain was in sequence (Letunic et al., 2002; Marchler-Bauer et al., 2002). Finally, the ExPASy^6^ ProtParam tool was used to query the physical and chemical properties of the *GmTIFYs* (Appel et al., 1994; Wang et al., 2020a).

### Phylogenetic Tree Analysis of TIFY Proteins

The TIFY protein sequences of *Arabidopsis*, rice, soybean, apple, and grape were compared using ClustalW in the MEGA-X software. The maximum likelihood (ML) method was used to construct a phylogenetic tree for analyzing the phylogenetic relationship between *TIFYs* (Kumar et al., 2016; Leng et al., 2021). The bootstrap method was used with 1,000 replicates. The methods and parameters were the same as the Jones–Taylor–Thornton (JTT) model, gamma-distributed rates (G), and the gamma parameter 1.

### Chromosomal Location, Gene Duplication, and Selective Pressure Analysis

The position information of the *GmTIFY* family genes was extracted from the GFF3 file of the soybean genome. The location and distribution of *GmTIFY* family genes were visualized on the chromosomes using Map Gene 2 Chromosomal (Jiangtao et al., 2015; Wang et al., 2020a).^7^

For gene duplication analysis, the TBtools software was used to identify the duplication events of the soybean genome and *GmTIFY* genes. The collinearity pairs of *GmTIFY* genes were extracted and used to visualize a synteny map with the CIRCOS software (Lestari et al., 2013).

The TIFY coding sequences were aligned using ClustalW software. The alignment results were converted to PAML format using EasyCodeML and a tree file in Newick format was built using MEGA-X. The selection pressure was estimated using the branch model of EasyCodeML. The ratio of non-synonymous to synonymous substitution rates (*ω*) was determined by the free-ratio model and the two-ratio model among the branches of the TIFY tree file (Gao et al., 2019).

### Gene Structure, Motif, and Promoter Sequence Analysis

The motif information of the soybean GmTIFY proteins was analyzed using the MEME online tool (Bailey et al., 2009; Wang et al., 2019).^8^ The resulting files and soybean gene structure annotation files were imported into TBtools for visualization.

The 2,000 bp promoter sequences were submitted to the PlantCARE website^9^ to analyze the *cis*-acting elements of its family members (Guo et al., 2007; Su et al., 2020; Wang et al., 2020a). The resulting file was imported into GSDS^10^ for visualization.

### Expression Patterns of *GmTIFY* Genes

The RNA-seq data of GmTIFY family members in different tissues and organs were downloaded from the Phytozome database.^11^ The transcriptome data of several abiotic stresses were obtained from previous studies (NCBI SRA accession: PRJNA694374; Shi et al., 2018). TBtools software was used to visualize the expression levels of GmTIFYs.

### Plant Materials, Stress Treatments, and Real-Time Fluorescence Quantitative PCR

The soybean variety Zhonghuang39 was used for this study. The soybeans were planted in a greenhouse in a mixture of humus and vermiculite (humus: vermiculite = 1:1). Seven-day-old soybean seedlings were treated with 10% PEG6000 and 250 mM NaCl, respectively. The samples were collected at 0, 0.5, 1, 2, 4, 8, 12, and 24 h after treatments (Li et al., 2017; Zhang et al., 2019).

An RNA plant extraction kit (Zhuangmeng, Beijing, China) was used to extract total RNA from soybean leaves and *Transcript*R All-in-One First-Strand cDNA Synthesis SuperMix (TransGen Biotech, Beijing, China) was used for reverse transcription. The primers designed by Primer Premier 5.0 software were listed in Supplementary Table 1. The eukaryotic elongation factor 1-β (*GmELF1b*) was used as the internal control (Jian et al., 2008). An Applied Biosystems 7500 Real-Time PCR System was used to perform RT-qPCR. The 2^−ΔΔ*C*T^ method was used to analyze the quantitative results analysis (Udvardi, 2008PC). Each experiment was performed with three biological replicates.

### Subcellular Localization Assay

Pectinase and cellulase were used to lyse fresh *Arabidopsis* leaves and obtain *Arabidopsis* protoplasts. The gene coding regions were cloned into the 16318hGFP expression vector. The fusion expression vector *GmTIFY10e*-hGFP and *GmTIFY10g*-hGFP were transformed into *Arabidopsis* protoplasts mediated by PEG4000, respectively (He et al., 2016). After 18 h of incubation at 22°C in the dark, the GFP fluorescence signal was detected using a laser confocal microscope (Zeiss LSM 700, Germany; Riechmann et al., 2000).

### Obtaining Transgenic *Arabidopsis* and Salt Stress Treatment

*Arabidopsis* (Col-0) seeds were sterilized with 75% alcohol for 15 min. Sterilized *Arabidopsis* seeds were sprinkled on ½ MS medium and maintained at 4°C for 4 days, after which they were moved to a growth incubator at 22°C under a 16 h light and 8 h dark cycle. When the seedlings grew to four leaves, they were transferred to a mixture of humus and vermiculite for subsequent experiments (Riechmann et al., 2000; Du et al., 2018).

The coding regions of the *GmTIFY* genes were subcloned into the pCAMBIA1302 vector. The constructed pCAMBIA1302-*GmTIFY10e* and pCAMBIA1302-*GmTIFY10g* were transformed into *Arabidopsis* using the floral dip method, respectively (Clough and Bent, 1998). Positive lines were selected on ½ MS medium plates containing hygromycin (35 mg/L) and were further verified using PCR. The same method was used until transgenic three generation (T_3_). The expression levels of transgenic lines were determined by RT-qPCR and three homozygous T_3_ lines with the highest expression levels were used for the subsequent phenotypic analysis (Li et al., 2017).

For the experiment of root growth, 5-day-old seedings were transferred to MS medium and MS medium with 125 mM NaCl for another 7 days, after which the lengths of primary root and fresh weights were measured (Wang et al., 2019). For salt treatment, 5-day-old seedlings were transferred to the soil, and then, 21-day-old seedlings were treated with 250 mM NaCl for 14 days (Wang et al., 2019). All experiments contained three independent replicates.

### Obtaining Soybean Hairy Roots by *Agrobacterium rhizogenes*-Mediated (*A. rhizogenes*-Mediated)

To obtain the overexpression vector of *GmTIFY* genes, the coding regions of *GmTIFY* genes were ligated with the pCAMBIA3301 vector to obtain recombinant plasmids (Kereszt et al., 2007; Zhao et al., 2017). To obtain the RNA interference expression vector, a 546 bp interference fragment consisting of a 200 bp target fragment and its antisense sequence connected by 146 bp zeol dehydrogenase gene sequence was synthesized and inserted into pCAMBIA3301 (Wang et al., 2019).

The constructed overexpression vector, interference expression vector, and empty pCAMBIA3301 vector were transferred to the *Bacillus rhizobacillus* (*B. rhizobacillus*) strain K599, and the recombinant vector was transferred to the hypocotyl of soybean *via* the *A. rhizogenes*-mediated method (Wang et al., 2015). The injected plants were cultured under high humidity conditions in a greenhouse until hairy roots grew at the infected site. When the hairy root reached about 5 cm long, the hypocotyl was removed below 0.5–1 cm of the infection site. At the same time, the seedlings were transplanted in mixed soil and cultured in the greenhouse for 7 days (Wang et al., 2020a). The positive soybean plants were subjected to salt stress test.

### Measurement of Physiological Indicators and Nitroblue Tetrazolium Staining

The leaves and roots of plants were used to determine physiological indicators under salt stress. A Physiological Index Test Kit (Cominbio, Suzhou, China) was used to test the contents of malondialdehyde (MDA), proline (PRO), catalase (CAT), and peroxidase (POD) in leaves and roots (Shi et al., 2018). The whole leaves and roots were soaked in nitroblue tetrazolium (NBT) for overnight staining. After staining, the samples were soaked in a decolorizing solution (30% glycerol and 70% ethanol) to decolorize the sample until it turned white (Du et al., 2018). All experiments were performed in three biological replicates.

### Enrichment Analysis of Co-expression Genes

The co-expression genes were obtained from the Phytozome database. The enrichment analysis was performed by Database for Annotation, Visualization and Integrated Discovery (DAVID) online tools.^12^ R software was used to visualize the results of the enrichment analysis.

### Statistical Analysis

One-way ANOVA test analysis was performed in Microsoft Excel 2007. Data were shown as means ± standard deviation (SD), with a *p*-value cutoff of 0.05 and 0.01. The method was used to analyze the RT-qPCR results and physiological indicators.

## Results

### Screening and Identification of *GmTIFY* Genes

The BLASTp program and hmmsearch program were used to search for *TIFY* genes in databases of *Arabidopsis*, rice, soybean, apple, and grape. We then compared the results of two programs and identified the TIFY domain using SMART and CDD to confirm TIFY members in five species. Finally, 38 *GmTIFY* genes were identified in the soybean genome. The number of *TIFY* genes in *Arabidopsis* (18), rice (20), grape (19), and apple (30) are consistent with previous reports (Ye et al., 2009; Bai et al., 2011; Li et al., 2014). The *GmTIFY* genes were named according to their relationship with *Arabidopsis* and their location on the chromosomes (Table 1). The protein lengths, molecular weights (MV), and isoelectric points (p*I*) are provided in Table 1. In 38 *GmTIFY* genes, the coding sequences range from 133 (*GmTIFY5d*) to 415 aa (*GmTIFY8a*); the MW ranges from 15.20 (*GmTIFY5d*) to 43.69 kD (*GmTIFY8a*) and the p*I* ranges from 4.63 to 9.89.

**Table 1:** Details of the 38 soybean *TIFY* genes.

Gene name	Gene ID	ORF (aa)	MW (kD)	Chromosome	p*I*
*GmTIFY1a*	Glyma_02G215600	310	33.34	2	6.08
*GmTIFY1b*	Glyma_04G096200	324	35.51	4	5.68
*GmTIFY1c*	Glyma_06G097900	304	33.44	6	5.70
*GmTIFY1d*	Glyma_14G182800	307	33.28	14	6.23
*GmTIFY2a*	Glyma_04G096300	350	37.87	4	4.63
*GmTIFY2b*	Glyma_06G098000	351	38.08	6	4.67
*GmTIFY2c*	Glyma_07G182700	355	39.59	7	4.91
*GmTIFY2d*	Glyma_08G067200	358	39.90	8	5.24
*GmTIFY2e*	Glyma_08G221800	334	36.69	8	6.56
*GmTIFY3a*	Glyma_09G077500	206	22.11	9	6.90
*GmTIFY3b*	Glyma_13G116100	207	22.71	13	9.89
*GmTIFY3c*	Glyma_15G184900	201	21.48	15	6.43
*GmTIFY3d*	Glyma_17G043700	197	21.47	17	9.79
*GmTIFY4a*	Glyma_10G244400	346	38.10	10	8.89
*GmTIFY4b*	Glyma_20G150000	350	38.31	20	8.89
*GmTIFY5a*	Glyma_05G141200	134	15.54	5	9.30
*GmTIFY5b*	Glyma_08G096500	150	17.34	8	9.81
*GmTIFY5c*	Glyma_13G219100	138	15.91	13	9.10
*GmTIFY5d*	Glyma_15G093100	133	15.20	15	8.68
*GmTIFY6*	Glyma_09G174200	386	41.54	9	9.44
*GmTIFY7a*	Glyma_05G235500	371	39.01	5	8.76
*GmTIFY7b*	Glyma_08G043000	369	39.39	8	8.98
*GmTIFY7c*	Glyma_09G123600	319	33.64	9	9.37
*GmTIFY8a*	Glyma_04G071400	415	43.69	4	7.28
*GmTIFY8b*	Glyma_06G072700	413	43.50	6	9.39
*GmTIFY8c*	Glyma_17G205200	379	40.53	17	6.83
*GmTIFY8d*	Glyma_08G264700	370	40.99	8	5.59
*GmTIFY8e*	Glyma_16G081800	392	43.29	16	5.75
*GmTIFY10a*	Glyma_01G204400	195	21.67	1	8.99
*GmTIFY10b*	Glyma_04G013800	201	21.93	4	8.75
*GmTIFY10c*	Glyma_06G013700	160	17.75	6	9.56
*GmTIFY10d*	Glyma_09G071600	258	27.83	9	8.83
*GmTIFY10e*	Glyma_11G038600	203	23.03	11	9.01
*GmTIFY10f*	Glyma_13G112000	242	26.29	13	7.74
*GmTIFY10g*	Glyma_15G179600	258	27.65	15	9.03
*GmTIFY10h*	Glyma_17G047700	242	26.36	17	9.00
*GmTIFY11a*	Glyma_07G041400	232	25.09	7	9.18
*GmTIFY11b*	Glyma_16G010000	230	24.90	16	9.00

### Phylogenetic Analysis of *GmTIFY* Genes

**Figure 1:**
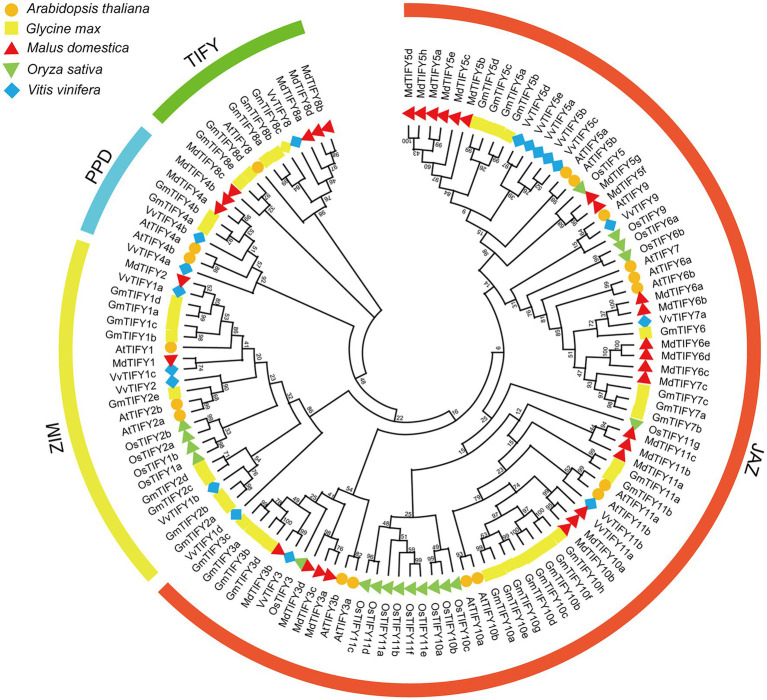
The phylogenetic tree of TIFY proteins from *Arabidopsis*, rice, grape, apple, and soybean. Multiple sequence alignment was performed by MEGA-X and the phylogenetic tree was constructed using the ML method. The TIFY, JAZ, PPD, and ZIM subfamilies are represented by different colors.

To investigate the phylogenetic relationships of GmTIFYs, we constructed a phylogenetic tree using *TIFY* genes from *Arabidopsis*, rice, soybean, grape, and apple (Figure 1). The *TIFY* genes were divided into four subfamilies of TIFY, JAZ, ZIM, and PPD according to specific domains (Zhang et al., 2012). The JAZ subfamily contains the largest number of *TIFY* genes including 12 *AtTIFY* genes, 11 *VvTIFY* genes, 22 *GmTIFY* genes, 22 *MdTIFY* genes, and 16 *OsTIFY* genes. The ZIM subfamily contains three *AtTIFY* genes, five *VvTIFY* genes, nine *GmTIFY* genes, two *MdTIFY* genes, and two *OsTIFY* genes. The PPD and TIFY subfamilies have no genes in monocotyledon plants which is consistent with the previous research results (Zhang et al., 2012; Li et al., 2014). There are two *AtTIFY* genes, two *VvTIFY* genes, two *GmTIFY* genes, and two *MdTIFY* genes in PPD subfamily and one *AtTIFY* genes, one *VvTIFY* genes, five *GmTIFY* genes, and four *MdTIFY* genes in TIFY subfamily. Further analysis revealed that the number of *TIFY* genes in soybeans and apples was almost twice than that of *Arabidopsis* and grapes. This may result from more events of chromosomes doubling, fusion and rearrangement occurred in soybean and apple evolution. Interestingly, we found the expression levels of *GmTIFYs* were significantly upregulated in our previous transcriptome data of drought, salt, and ABA treatments which were all *TIFY10* and *TIFY11* homologous genes. The transgenic *Arabidopsis* lines of *AtTIFY10a* and *AtTIFY10b* showed higher salt tolerance than WT plants (Zhu et al., 2014). We speculated that the *GmTIFY10* and *GmTIFY11* homologous genes may be involved in responding to salt stress.

### Chromosomal Location and Gene Duplication

**Figure 2:**
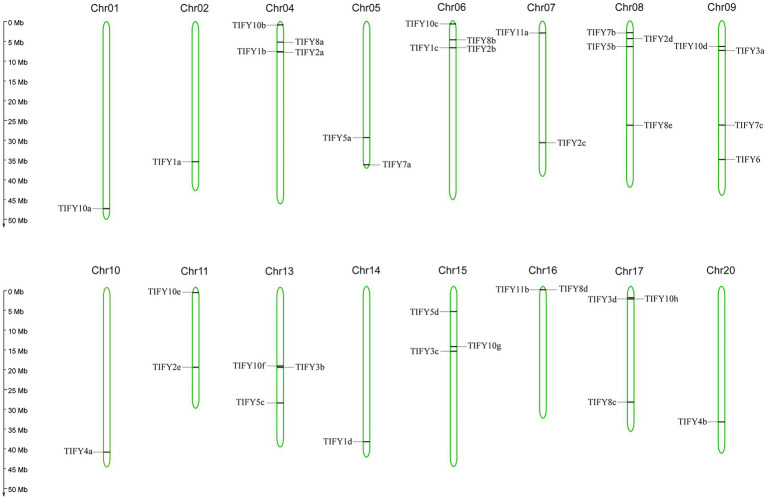
The location of 38 *GmTIFY* genes on the soybean chromosome. The scale bar on the left indicates the size of the chromosomes.

**Figure 3:**
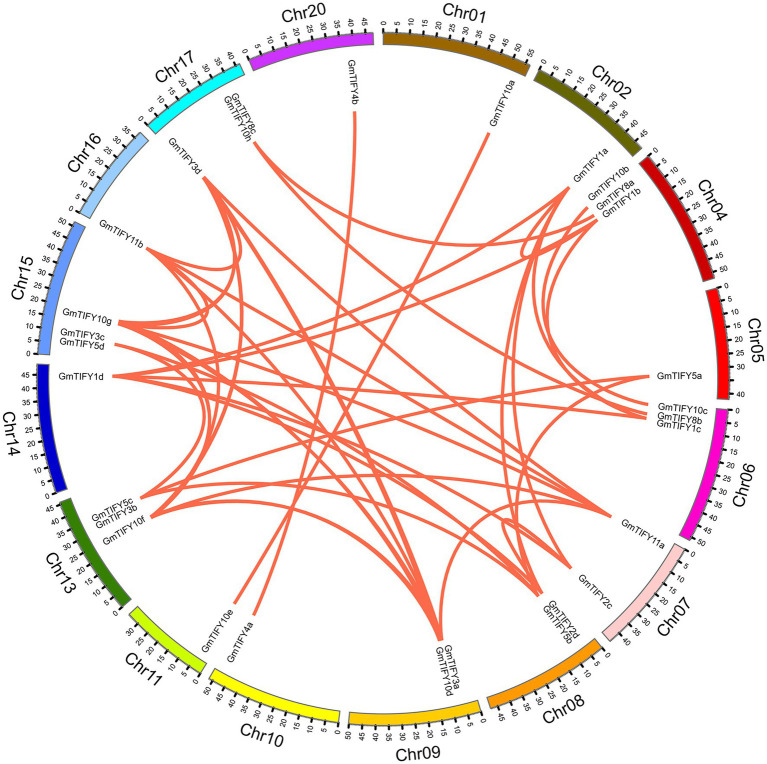
Distribution of segmentally duplicated *GmTIFY* genes on soybean chromosomes. Red lines indicate duplicated *GmTIFY* gene pairs.

In the soybean genome, 38 *GmTIFY* genes are irregularly distributed on 16 chromosomes (Figure 2). The duplication events analysis demonstrated that nine *GmTIFY* genes were singletons and 29 *GmTIFY* genes experienced gene duplication events, including eight genes with segmental duplication and 21 genes with dispersed duplication (Figure 3). The most duplication events occurred in the JAZ subfamily (29 times). Only one duplication event occurred in the PPD subfamily. Further analysis demonstrated that large segmental chromosome duplication events occurred between chromosomes 4/6 and chromosomes 10/20. The most duplication events were identified in the *TIFY10* homologous genes in the JAZ subfamily (15 times).

To determine the significance of *GmTIFY* family genes during evolution, the EasyCodeML software was used to test the selective pressure of *GmTIFY* genes including purification selection, positive selection, and negative selection (Table 2). Since the *TIFY1* and *TIFY2* homologous genes are always grouped together in the evolutionary tree, we calculated their total *ω*. The positive selection of *GmTIFY1*, *GmTIFY2*, *GmTIFY3*, *GmTIFY7*, and *GmTIFY10* homologous genes exceeded 1, which indicated that these *TIFY* homologous genes experienced positive selection during their evolutionary history.

**Table 2:** Analysis of natural selection patterns using PAML.

Subfamily	Group	Model	LnL	Estimates of Parameters	
				Background (*ω*)	Foreground (*ω*)
ZIM	1 + 2	Two-ratio Model 2	−1,672.124876	0.60068	1.28945
		Model 0	−1,672.422571	0.61229	
JAZ	3	Two-ratio Model 2	−1,672.472447	0.61143	1.59695
		Model 0	−1,672.422571	0.61229	
	4	Two-ratio Model 2	−1,672.402008	0.61570	0.47849
		Model 0	−1,672.422571	0.61229	
	5	Two-ratio Model 2	−1,672.420049	0.61160	0.68454
		Model 0	−1,672.422571	0.61229	
	6	Two-ratio Model 2	−1,672.411868	0.60902	0.66408
		Model 0	−1,672.422571	0.61229	
	7	Two-ratio Model 2	−1,672.422571	0.61229	1.47477
		Model 0	−1,672.422571	0.61229	
TIFY	8	Two-ratio Model 2	−1,672.417476	0.60916	0.74566
		Model 0	−1,672.422571	0.61229	
JAZ	10	Two-ratio Model 2	−1,672.422575	0.61229	1.65538
		Model 0	−1,672.422571	0.61229	
	11	Two-ratio Model 2	−1,672.422571	0.61230	0.61216
		Model 0	−1,672.422571	0.61229	

### Gene Structure, Motif Composition, and *cis*-Element Analysis of *GmTIFY* Genes

**Figure 4:**
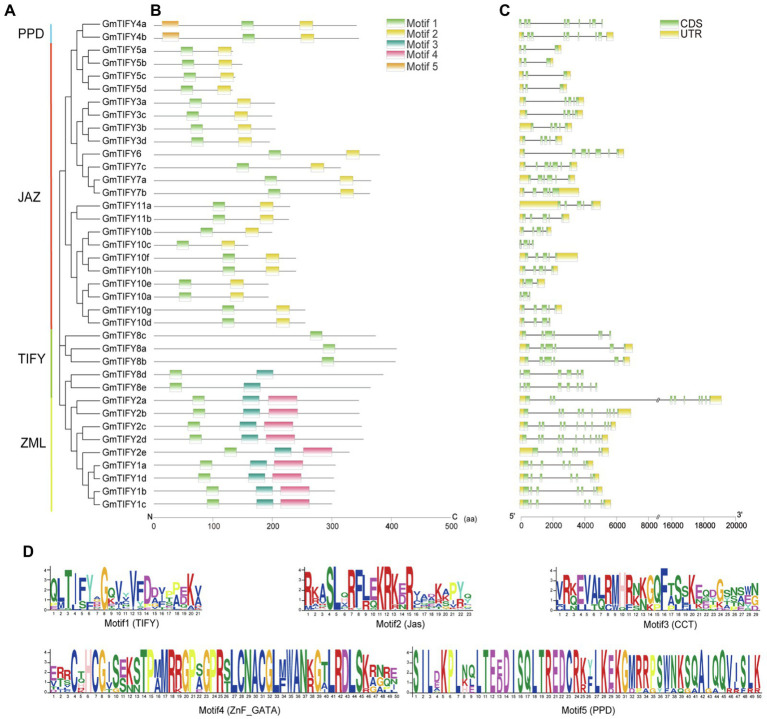
Phylogenetic relationships, conserved motifs, and gene structures of GmTIFY. **(A)** Phylogenetic tree of TIFY proteins from soybean constructed using the ML method. **(B)** Distribution of conserved motifs in GmTIFY proteins. Five putative motifs are indicated by colored boxes. **(C)** Exon/intron organization of *GmTIFY* genes. Green boxes represent exons and black lines represent introns. The upstream/downstream regions of *GmTIFY* genes are indicated by yellow boxes. The scale at the bottom is used to infer exons length. **(D)** Motif logos of five conservative motifs detected in GmTIFY proteins *via* MEME analysis.

The structural characteristics of *GmTIFY* family genescan be obtained by analyzing the phylogenetic tree, motifs, and positions of exons and introns (Figure 4). These results showed that genes belonging to the same phylogenetic group have similar motifs and exon/intron structures. According to previous studies of *TIFY* genes, *GmTIFY* family genes were divided into four subfamilies (TIFY, JAZ, PPD, and ZML) with different structural features. Five conserved motifs were found based on the analysis of TIFY protein sequences (Figure 4B). Motif 1 was identified as TIFY domain and distributed in all genes. Motif 2 (Jas domain) was contained by the JAZ and PPD subfamily genes. Motif 3 and 4 were identified as the sequence of CCT domain and the ZnF_GATA domain in ZIM subfamily, respectively. The PPD subfamily contains motif 1, 2, and 5, while motif 2 of the PPD subfamily lacks a PY motif which different from the original Jas domain. There was no PPD motif sequence information in the Pfam database. The conservative motif 5 domain sequence was constructed with PPD subfamily genes which were previously annotated in *Arabidopsis*, apples, and grapes. The motif 5 was confirmed with sequences from the report of earliest defined PPD genes (Bai et al., 2011). Conserved sequences were submitted to the MEME online tool to generate the domain logo (Figure 4D). Analysis of the promoters through PlantCARE website revealed that *GmTIFY* family genes contain many abiotic stresses responsive *cis*-elements, such as ABA-responsive element (ABRE), MYB banding site (MBS), and methyl jasmonate-responsive element (MeJA element) (Supplementary Figure 1).

### Expression Patterns of *GmTIFY* Genes

**Figure 5:**
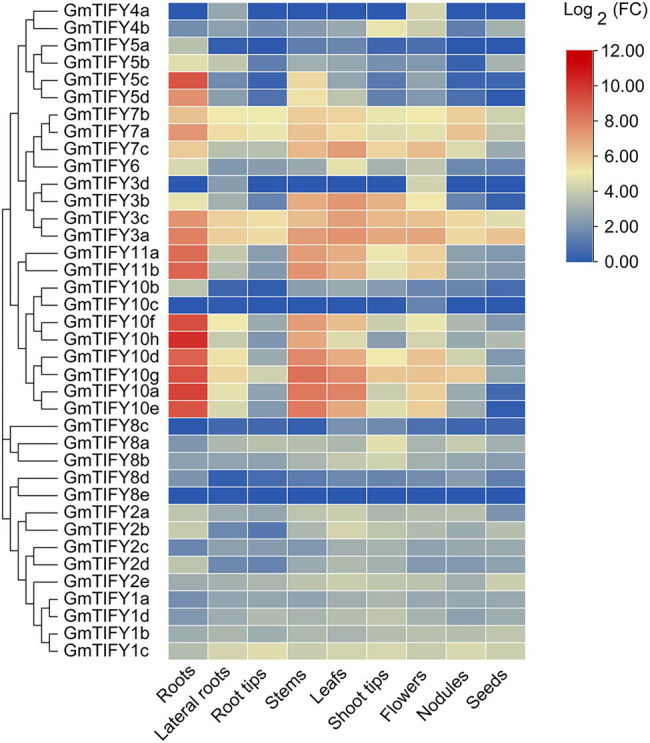
The expression profiles of 38 *GmTIFY* genes in different soybean tissues. The expression data of 38 *GmTIFY* genes in different tissues were obtained from the Phytozome database.

To obtain the expression profiles of soybean *GmTIFY* genes in different tissues, RNA-seq data were downloaded from the Phytozome database and visualized by using the TBtools software (Figure 5). Results demonstrated that *GmTIFY* genes had significant expression differences in multiple tissues. The expression levels of most genes are relatively low in all tissues or are not even expressed. The homologous genes of *TIFY3*, *TIFY5*, *TIFY10*, and *TIFY11* in the JAZ subfamily showed higher expression in the roots, stems, and leaves.

**Figure 6:**
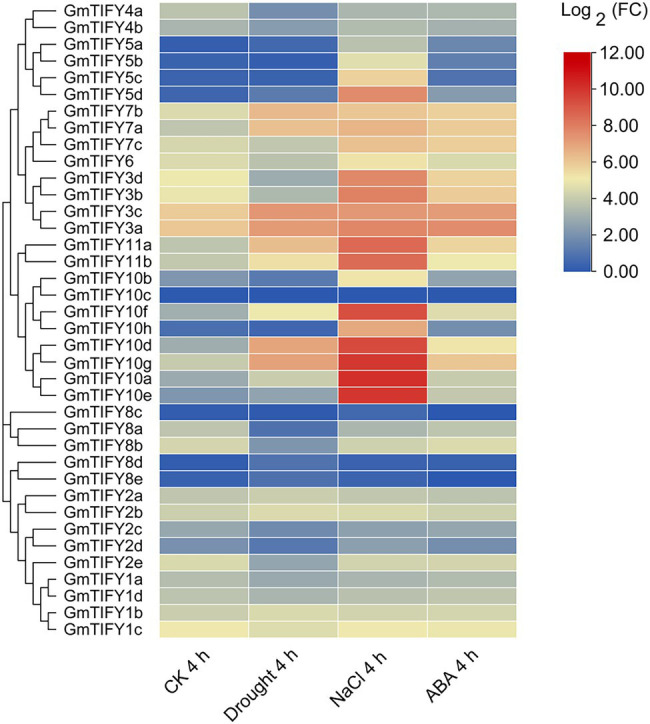
The expression profiles of 38 *GmTIFY* genes under different conditions including drought, salt and ABA treatments.

**Figure 7:**
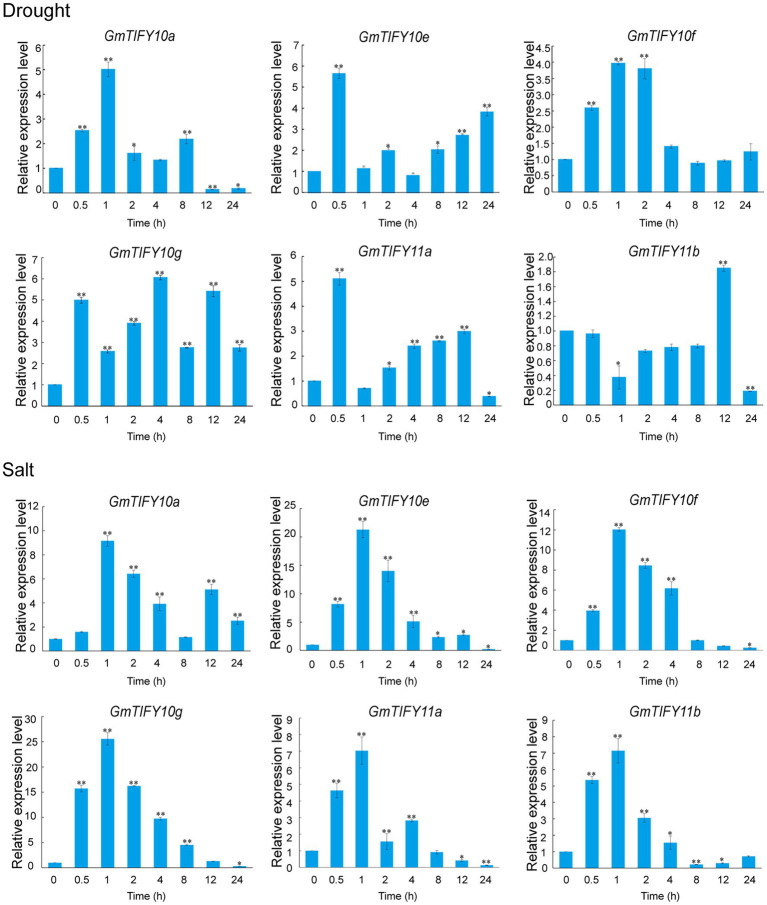
Expression patterns of *GmTIFY* genes under drought and salt stress conditions. Expression levels of six *GmTIFY* genes were measured using RT-qPCR at different times under drought and salt treatments. RT-qPCR data were normalized using *GmELF1b* as the reference gene and were displayed relative to 0 h. The *x*-axes show the duration of treatment, and the y-axes depict relative expression levels (error bars indicate SD). The data are shown as the means of three biological replicates ±SD. ANOVA test demonstrates that there are significant differences (^*^p < 0.05, ^**^p < 0.01).

We analyzed the transcriptome data of *GmTIFY* genes under drought, salt and ABA treatment (Figure 6). Most *TIFY10* and *TIFY11* homologous genes significantly responded to salt treatment. From these homologous genes, six significantly upregulated candidate genes (*GmTIFY10a*, *GmTIFY10e*, *GmTIFY10f*, *GmTIFY10g*, *GmTIFY11a*, and *GmTIFY11b*) were selected and confirmed their expression level under drought and salt stress by RT-qPCR (Figure 7). The six genes were not significantly changed after drought treatment. After salt stress, the expression levels of six genes were significantly upregulated (>10-fold), especially *GmTIFY10e* and *GmTIFY10g*, which reached the peak at 1 h.

### *GmTIFY10e* and *GmTIFY10g* Are Localized in the Nucleus

**Figure 8:**
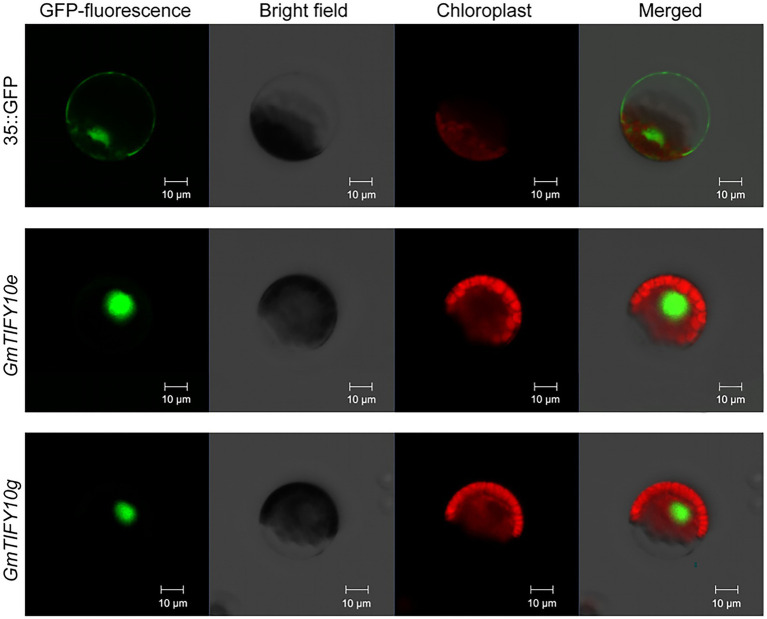
Subcellular localization of *GmTIFY10e*-hGFP and *GmTIFY10g*-hGFP fusion protein. 35S::16318hGFP was used as a control. The scale bar of 35S::16318hGFP, *GmTIFY10e*-hGFP and *GmTIFY10g*-hGFP indicates 10 μm.

The results of the phylogenetic, duplication events, selective pressure, and expression profiles of *GmTIFY* genes indicated that *GmTIFY10e* and *GmTIFY10g* could play an important role in soybean. We selected *GmTIFY10e* and *GmTIFY10g* for further study, both of which significantly responded to salt stress. To determine the subcellular localization of *GmTIFY10e* and *GmTIFY10g*, we transformed the recombinant 16318hGFP vector linked to the *GmTIFY10e* and *GmTIFY10g* into *Arabidopsis* protoplasts using the PEG4000-mediated method, respectively (Figure 8). Both *GmTIFY10e*-hGFP and *GmTIFY10g*-hGFP fusion proteins are located in the nucleus.

### *GmTIFY10e* and *GmTIFY10g* Can Improve Salt Tolerance in Transgenic *Arabidopsis*

**Figure 9:**
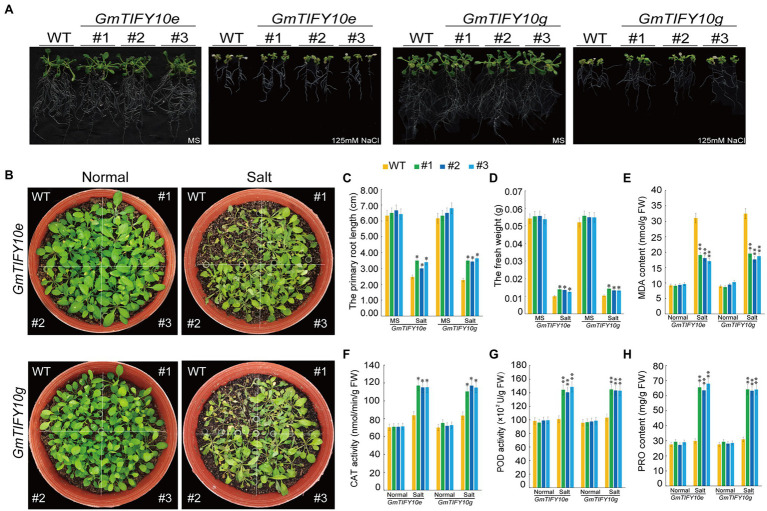
Overexpression of *GmTIFY10e* and *GmTIFY10g* in *Arabidopsis* plants enhanced tolerance to salt stress. **(A)** Phenotypic analysis of transgenic *Arabidopsis* lines and WT lines under normal and 125 mM NaCl treatment. The scale bar indicates 2 cm. **(B)** Phenotypes of WT and transgenic plants under salt stress. **(C)** The primary root length analysis of the transgenic *Arabidopsis* lines and WT plants under normal and 125 mM NaCl treatment. **(D)** The fresh weights of transgenic *Arabidopsis* lines and WT lines under normal and 125 mM NaCl conditions. **(E–H)** The leaf contents of MDA **(E)**, CAT **(F)**, POD **(G),** and PRO **(H)** in WT and transgenic plants under normal and salt stress. The data are shown as the means ± SD obtained from three biological replicates. ANOVA test demonstrates significant differences compared with WT (^*^p < 0.05, ^**^p < 0.01).

To investigate the function of *GmTIFY10e* and *GmTIFY10g* in plant salt tolerance, we obtained transgenic *Arabidopsis* lines with high expression levels of *GmTIFY10e* and *GmTIFY10g*, respectively. For root length assay, the 5-day-old *Arabidopsis* plants were transferred to MS medium containing 125 mM NaCl and we calculated the primary root lengths and fresh weights. Under normal conditions, there was no significant difference between the WT plants and transgenic lines. However, the growth of the WT plants was significantly repressed when treated with 125 mM NaCl and the transgenic lines showed better growth than WT plants, with longer root lengths and heavier fresh weights (Figures 9A,C,D). To verify the salt tolerance of plants in soil, 21-day-old plants were subjected to salt stress (Figure 9B). There is no significant difference between the transgenic and WT plants under normal growth conditions. After 14 days of 250 mM NaCl treatment, the transgenic lines showed higher salt tolerance than WT, when WT is seriously wilted or even dead.

Salt stress can reduce the scavenging function of reactive oxygen species (ROS) in cells (Raza et al., 2021). Accumulation of ROS will lead to membrane lipid peroxidation, forming MDA, and activating oxygen enzymatic scavenging system including CAT, POD (Mhamdi and Van Breusegem, 2018; Wang et al., 2021a; Youssef et al., 2021). At the same time, plant cells will accumulate a large number of PRO under stress to maintain normal cell swelling pressure, prevent excessive water loss of protoplasm and enhance the adaptability of plants to adversity (Wang et al., 2021b). We measured the MDA, PRO, CAT, and POD contents of transgenic and WT lines under normal and salt treatment conditions (Figures 9E–H). Compared with WT plants, the contents of PRO, CAT, and POD in the transgenic lines were significantly increased, while the MDA contents of the transgenic lines were significantly reduced. These results all indicated that *GmTIFY10e* and *GmTIFY10g* play a role in improving the tolerance of salt stress.

### Overexpression of *GmTIFY10e* and *GmTIFY10g* Improve Salt Tolerance in Soybean Hairy Roots

**Figure 10:**
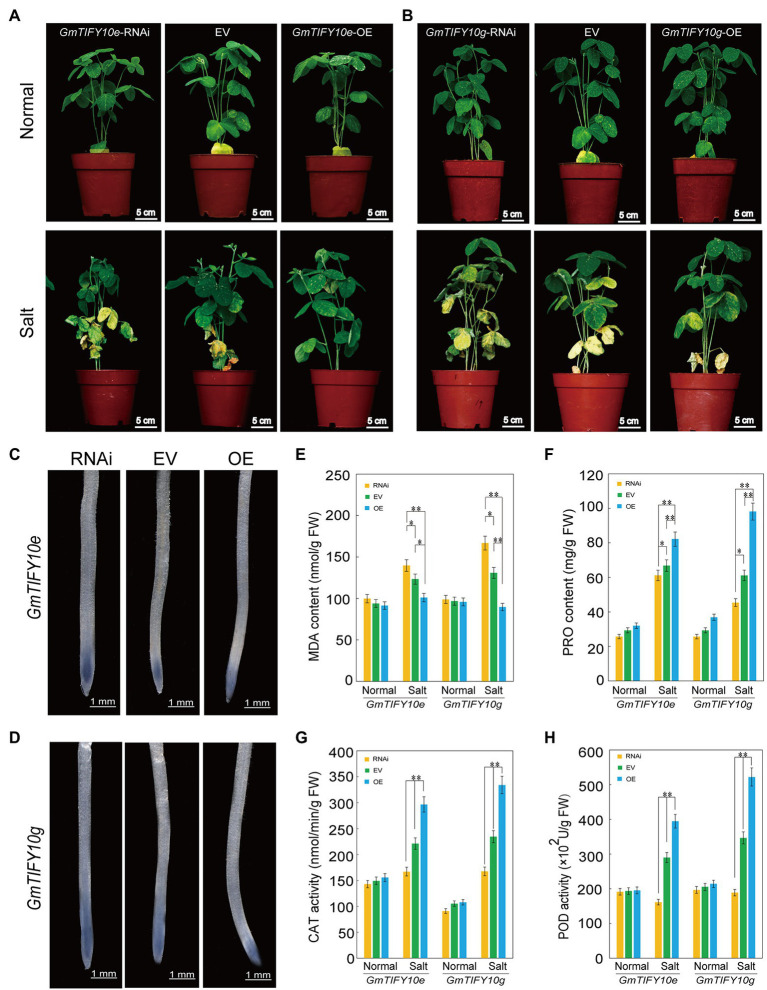
Analysis of the function of soybean *GmTIFY10e* and *GmTIFY10g* under normal and salt stress. **(A,B)** Phenotype analysis of EV and transgenic plants under normal and salt stress. The scale bar indicates 5 cm. **(C,D)** NBT staining of EV and transgenic plant roots under salt stress. The scale bar indicates 1 mm. **(E–H)** The MDA **(E)**, PRO **(F)**, CAT **(G)**, and POD **(H)** contents of EV and transgenic plant leaves under normal and salt stress. The data are shown as the means ± SD obtained from three biological replicates. ANOVA test demonstrates that there are significant differences compared with EV (^*^p < 0.05, ^**^p < 0.01).

To verify the function of *GmTIFY10e* and *GmTIFY10g* in response to salt stress in soybean, we obtained overexpressing plants (OE), empty vector plants (EV) and RNAi plants with *A. rhizogenes*-mediated transformation of soybean hairy roots and treated with 250 mM NaCl. RT-qPCR analysis demonstrated that the expression levels of OE plants were significantly higher than that of the EV, while the expression levels of RNAi plants were lower than that of the EV (Supplementary Figure 3). Phenotypic identification showed that there was no significant difference among the OE plants, RNAi plants, or EV plants under normal growth conditions. After 5 days treatment of salt, the leaves of the RNAi plants turned yellow and were more wilted than the EV plants. In the OE lines, only a few bottom leaves showed yellowing and wilting, which indicated significantly increased salt tolerance (Figures 10A,B). It indicated that overexpression of *GmTIFY10e* and *GmTIFY10g* improved the salt stress tolerance in soybean.

Reactive oxygen species is the most important signal substance for plants to respond to abiotic stresses. A large amount of ROS will accumulate in plants under abiotic stresses. NBT staining can reveal the levels of ROS accumulation in plants (Figures 10C,D). We measured the levels of ROS accumulation of plants roots. After salt treatment, the staining levels of OE plants were significantly lighter than that of EV plants and the RNAi plants showed more significant NBT stains. The same results were obtained from staining the leaves of OE plants, RNAi plants, and EV plants (Supplementary Figures 4A,F).

We further measured the contents of MDA, PRO, CAT, and POD in the roots of OE plants, RNAi plants and EV plants (Figures 10E–H). Under normal growth conditions, the MDA, PRO, CAT, and POD contents in OE plants and RNAi plants were not significantly different than in EV plants. After salt treatment, the PRO, POD, and CAT contents in OE plants were significantly higher than in EV plants and RNAi plants, while the MDA content in OE plants was lower than in EV plants and RNAi plants. We obtained similar results by measuring the MDA, PRO, CAT, and POD contents of the leaves (Supplementary Figures 4C–F). These results further confirmed that overexpression of *GmTIFY10e* and *GmTIFY10g* can enhance salt stress tolerance in soybean, which is consistent with the results of transgenic *Arabidopsis*.

### Overexpression of *GmTIFY10e* and *GmTIFY10g* Can Influence the Expression Levels of ABA-Related Genes

**Figure 11:**
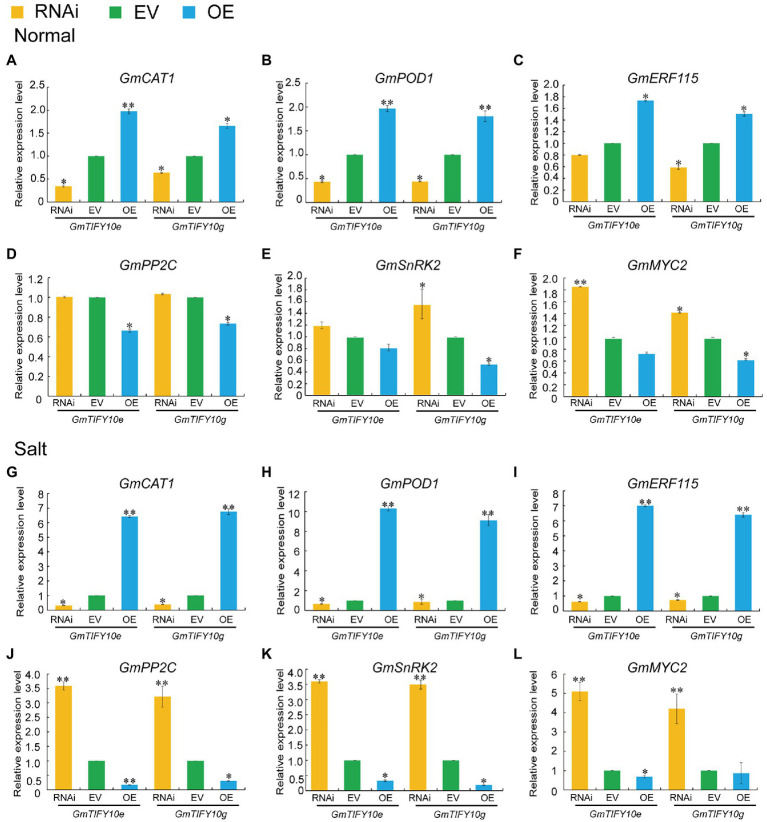
**(A–F)** The GmCAT1 **(A)**, GmPOD **(B)**, GmERF115 **(C)**, GmPP2C **(D)**, GmSnRK2 **(E)** and GmMYC2 **(F)** expression levels of EV and transgenic plants under normal conditions. **(G–L)** The GmCAT1 **(G)**, GmPOD **(H)**, GmERF115 **(I)**, GmPP2C **(J)**, GmSnRK2 **(K)** and GmMYC2 **(L)** expression levels of EV and transgenic plants under salt stress. Vertical bars indicate ±SD of three replicates. ANOVA test demonstrates that there are significant differences compared with EV (^*^p < 0.05, ^**^p < 0.01).

Previous transcriptome data demonstrated that the expression levels of *GmTIFY10e* and *GmTIFY10g* were upregulated under ABA treatment. Enrichment analysis of *GmTIFY10e* and *GmTIFY10g* co-expression genes demonstrated that *GmTIFY10e* and *GmTIFY10g* can participate in the JA signal pathway and the ABA signal pathway (Supplementary Figure 5). In previous studies, the JAZ proteins were identified to participate in the ABA-dependent signal pathway through the target protein *MYC*/*MYB* transcription factor or ABA signal receptor *PYL4* (Fu et al., 2017; Luo et al., 2020). To study the possible mechanisms regulated by *GmTIFY10e* and *GmTIFY10g* during salt stress responses, we analyzed the expression levels of *GmCAT1*, *GmPOD*, and *GmERF115* which were genes in the ABA-mediated osmotic stress signals, and the *GmSnRK2*, *GmPP2C*, and *GmMYC2* which were key genes in the ABA signal transduction pathway (Figure 11). Under normal growth conditions, the expression levels of *GmCAT1*, *GmPOD*, and *GmERF115* genes were higher in OE plants and were lower in RNAi plants compared to EV plants, while *GmSnRK2*, *GmPP2C*, and *GmMYC2* were higher in RNAi plants. For salt stress, the expression levels of *GmCAT1*, *GmPOD*, and *GmERF115* were significantly upregulated in OE plants and downregulated in RNAi plants, while *GmSnRK2*, *GmPP2C*, and *GmMYC2* significantly upregulated in RNAi plants and downregulated in OE plants. These results indicated that *GmTIFY10e* and *GmTIFY10g* may be involved in responding to salt stress through the ABA regulation pathway.

## Discussion

As a kind of specific plant proteins, TIFY proteins play an important role in the plant growth and response to environmental changes. However, the information about the expression and function of TIFY family in soybean are very limited. This study systematically identified and analyzed 38 *GmTIFY* genes and their responses to abiotic stresses in soybean. Our results confirmed that soybean *GmTIFY10e and GmTIFY10g* genes can positively regulate the salt stress tolerance in plants. *AtTIFY10a* and *AtTIFY10b* significantly improved tolerance to salt and alkali stresses in *Arabidopsis* plants (Zhu et al., 2014). In cotton, overexpression of *TIFY10* homologous genes *GaJAZ1* could significantly improve the tolerance to salt stress. In addition, the *GaJAZ1* was confirmed to interact with *GaMYC2* to repress expression of downstream genes related to ABA signaling pathways, affecting plant tolerance to salinity stress. Therefore, *TIFY10* homologous genes may be involved in abiotic stresses tolerance, including salt tolerance (Zhao et al., 2020b).

ABA and ABA signaling pathway play important roles in regulating various stress responses (Stone et al., 2006; Nilson and Assmann, 2007; Sirichandra et al., 2009). ABA-related *cis*-elements can combine with transcription factors to regulate the expression of corresponding genes and regulate the sensitivity of plants to ABA signaling pathway (Kim et al., 2011). *GmTIFY10e* and *GmTIFY10g* have several ABA-related elements and its co-expression genes were involved in the ABA-activated signaling pathway and the JA signaling pathway (Supplementary Figures 1, 5). Transcriptome data demonstrated that the expression levels of *GmTIFY10e* and *GmTIFY10g* were upregulated under ABA treatment (Figure 6). These results indicated that these two genes may be related to ABA signaling pathway.

The tolerance of ABA to environmental stresses mainly depends on antioxidant protection system (Wu et al., 2021). Abiotic stresses can induce the production of H_2_O_2_ in plants. H_2_O_2_ can directly act on the negative regulatory factor *PP2Cs* of ABA signaling pathway and promote the expression of *CAT1* and *POD* genes. The *AtERF115* can mediate the ROS pathway and maintain the root stem and root growth through phytosulfokine (PSK) peptide incorporation (Kong et al., 2018). *GmTIFY10e* and *GmTIFY10g* could significantly affect the expression levels of *GmCAT1*, *GmPOD*, and *GmERF115* genes under salt stress (Figures 11A–C, G–I). To further analyze the regulation mechanism of *GmTIFY10e* and *GmTIFY10g*, we measured the key genes of ABA signal transduction pathway, which mainly includes *PP2Cs*, *SnRK2s*, and *MYCs*. The JAZ protein participated in the ABA-dependent signal pathway through its target protein *MYC*/*MYB* transcription factor and can interact with the ABA signal receptor *PYL4*. In presence of ABA, it will combine with *PYLs* and inhibit the phosphatase of *PP2Cs* and inhibit the *SnRK2s* (Lackman et al., 2011; Fu et al., 2017; Luo et al., 2020; Wang et al., 2020b). Our results showed that overexpression of *GmTIFY10e* and *GmTIFY10g* in soybean could significantly decrease the expression levels of *GmSnRK2*, *GmPP2C*, and *GmMYC2* genes compared with EV plants under salt stress (Figures 11J–L). Therefore, *GmTIFY10e* and *GmTIFY10g* maybe affect the salt stress tolerance through ABA pathway in plants.

## Conclusion

We identified 38 *GmTIFY* genes in soybean genome, among which *GmTIFY10e* and *GmTIFY10g* were significantly upregulated by salt stress. Overexpression of *GmTIFY10e* and *GmTIFY10g* could improve the salt tolerance of transgenic plants by inhibiting the expression of key genes of ABA pathway. This research provides a basis for further study on how TIFY family members affect salt tolerance.

## Data Availability Statement

The original contributions presented in the study are included in the article/Supplementary Material, and further inquiries can be directed to the corresponding authors.

## Author Contributions

Z-SX coordinated the project, conceived and designed the experiments, and edited the manuscript. Y-LL performed the experiments and wrote the first draft. LZ, Z-SX, and J-HL revised the manuscript. L-GJ, Y-XL, Y-NK, Y-XW, T-FY, JC, Y-BZ, F-ZW, and MC contributed to data analysis and managed reagents. Y-ZM and J-HL contributed with valuable discussions. All authors contributed to the article and approved the submitted version.

## Funding

This research was financially supported by the National Natural Science Foundation of China (31871624), the Agricultural Science and Technology Innovation Program (CAAS-ZDRW202109 and CAAS-ZDRW202002), Chinese Academy of Agricultural Sciences and the central government of Shandong Province guides the development of local science and technology (YDZX20203700002548), and Key R&D Projects of Shandong Province (2021LZGC014).

## Conflict of Interest

The authors declare that the research was conducted in the absence of any commercial or financial relationships that could be construed as a potential conflict of interest.

## Publisher’s Note

All claims expressed in this article are solely those of the authors and do not necessarily represent those of their affiliated organizations, or those of the publisher, the editors and the reviewers. Any product that may be evaluated in this article, or claim that may be made by its manufacturer, is not guaranteed or endorsed by the publisher.

## Footnotes

^1^ http://plants.ensembl.org/index.html

^2^ http://pfam.Xfam.org/

^3^ http://www.arabidopsis.org/

^4^ http://smart.embl-heidelberg.de/

^5^ https://www.ncbi.nlm.nih.gov/Structure/cdd/wrpsb.cgi

^6^ https://www.expasy.org/

^7^ http://mg2c.iask.in/mg2c_v2.0/

^8^ http://meme-suite.org/

^9^ http://bioinformatics.psb.ugent.be/webtools/plantcare/html/

^10^ http://gsds.cbi.pku.edu.cn/

^11^ https://phytozome-next.jgi.doe.gov/pz/portal.html

^12^ https://david.ncifcrf.gov/
