# Supplementary material for: Genome-Wide Analysis of the Soybean TIFY Family and Identification of GmTIFY10e and GmTIFY10g Response to Salt Stress
Source: Front Plant Sci. 2022 Mar 23;13:845314. doi: 10.3389/fpls.2022.845314 (PMC8984480; doi:10.3389/fpls.2022.845314)
Supplement: Supplementary file 1 [file Data_Sheet_1.docx]

**Genome-wide Analysis of the Soybean TIFY Family and Identification of *GmTIFY10e* and *GmTIFY10g* Response to Salt Stress**

**Running title: *GmTIFY10e and GmTIFY10g Genes* confers stress tolerance in transgenic plant**

Ya-Li Liu^1,2†^, Lei Zheng^2†^, Long-Guo Jin^2†^, Yuan-Xia Liu^1^, Ya-Nan Kong^1^, Yi-Xuan Wang^1^, Tai-Fei Yu^2^, Jun Chen^2^, Yong-Bin Zhou^2^, Ming Chen^2^, Feng-Zhi Wang^3^, You-Zhi Ma^2^, Zhao-Shi Xu^2^* and Jin-Hao Lan^1^*

^1^ College of Agronomy, Qingdao Agricultural University, Qingdao 266109, China.

^2^ Institute of Crop Science, Chinese Academy of Agricultural Sciences (CAAS)/National Key Facility for Crop Gene Resources and Genetic Improvement, Key Laboratory of Biology and Genetic Improvement of Triticeae Crops, Ministry of Agriculture, Beijing 100081, China.

^3^ Hebei Key Laboratory of Crop Salt-alkali Stress Tolerance Evaluation and Genetic Improvement/Cangzhou Academy of Agriculture and Forestry Sciences, Cangzhou 061000, China.

† These authors contributed equally to this work.

* Corresponding author: xuzhaoshi@caas.cn and [jinhao2005@qau.edu.cn](mailto:jinhao2005@qau.edu.cn)

This file includes:

Supplementary Table 1

Supplementary Figure 1 to 5

Supplementary Table 1. The primers used for the experiments.

For RT-qPCR

| Name | Sequence (5'-3') |
| --- | --- |
| GmELF1b-F | GTTGAAAAGCCAGGGGACA |
| GmELF1b-R | TCTTACCCCTTGAGCGTGG |
| AtActin-F | GCATGAAGATCAAGGTGGTTGCAC |
| AtActin-R | ATGGACCTGACTCATCGTACTCACT |
| GmTIFY10a-F | CCTCCTTATCGCTTGGTA |
| GmTIFY10a-R | GCACAGTCGTTCTGGTTT |
| GmTIFY10e-F | AACATGGGAAATTCCAGTGTTG |
| GmTIFY10e-R | GGCACTGGTTGGAATGATATTC |
| GmTIFY10f-F | CCAAAGCACCATATCAACTAGC |
| GmTIFY10f-R | TAGAGCAGCATCAACTAACCAA |
| GmTIFY10g-F | CATGAACTTGTTTCCTCCCAAG |
| GmTIFY10g-R | AAGGTTGGAATCTCCTCTGAAG |
| GmTIFY11a-F | CCAAGGAGAGCTTCACTTCTAA |
| GmTIFY11a-R | GGCTTGAGATTGTTCATTTGGT |
| GmTIFY11b-F | CAGTTCTTGAAGGAGAAACGTG |
| GmTIFY11b-R | TCCTAACAAACCCTTGGTACTC |
| GmCAT1-F | GAACAACTTCAAGCAGCCCG |
| GmCAT1-R | GCCTCGTGCTGAGATGAGAA |
| GmPOD-F | ACATTGGAGTGCTAACGGGA |
| GmPOD-R | TGAGCTAACCATGCCATCTGA |
| GmERF115-F | CACCTCACAAATCAGCACAGC |
| GmERF115-R | TGTTACTCCCACCACCCATGA |
| GmMYC2-F | TTCCTTCACTTCCCTGCC |
| GmMYC2-R | CTTTTCTCTCCTCTGTCTCTCG |
| GmPP2C-F | ATCTTGGGCGTGGTGGA |
| GmPP2C-R | TGTTAGGCTCATGGTCTGTAGTC |
| GmSnRK2-F | CTATAAAGATCCTTAACCGCCA |
| GmSnRK2-R | GCTCTCCAGATTTCACATACTCC |

For cloning

| Name | Sequence (5'-3') |
| --- | --- |
| GmTIFY10e-F | ATGAATCCATGGAACATGAGAACTT |
| GmTIFY10e-R | CTAAAATAACACAAAGCTGGAGCTGC |
| GmTIFY10g-F | ATGTCGAGCTCATCGGAGTAC |
| GmTIFY10g-R | TCAGATTTGAGGTGAAGAAGCAC |

**Supplementary Figure 1**


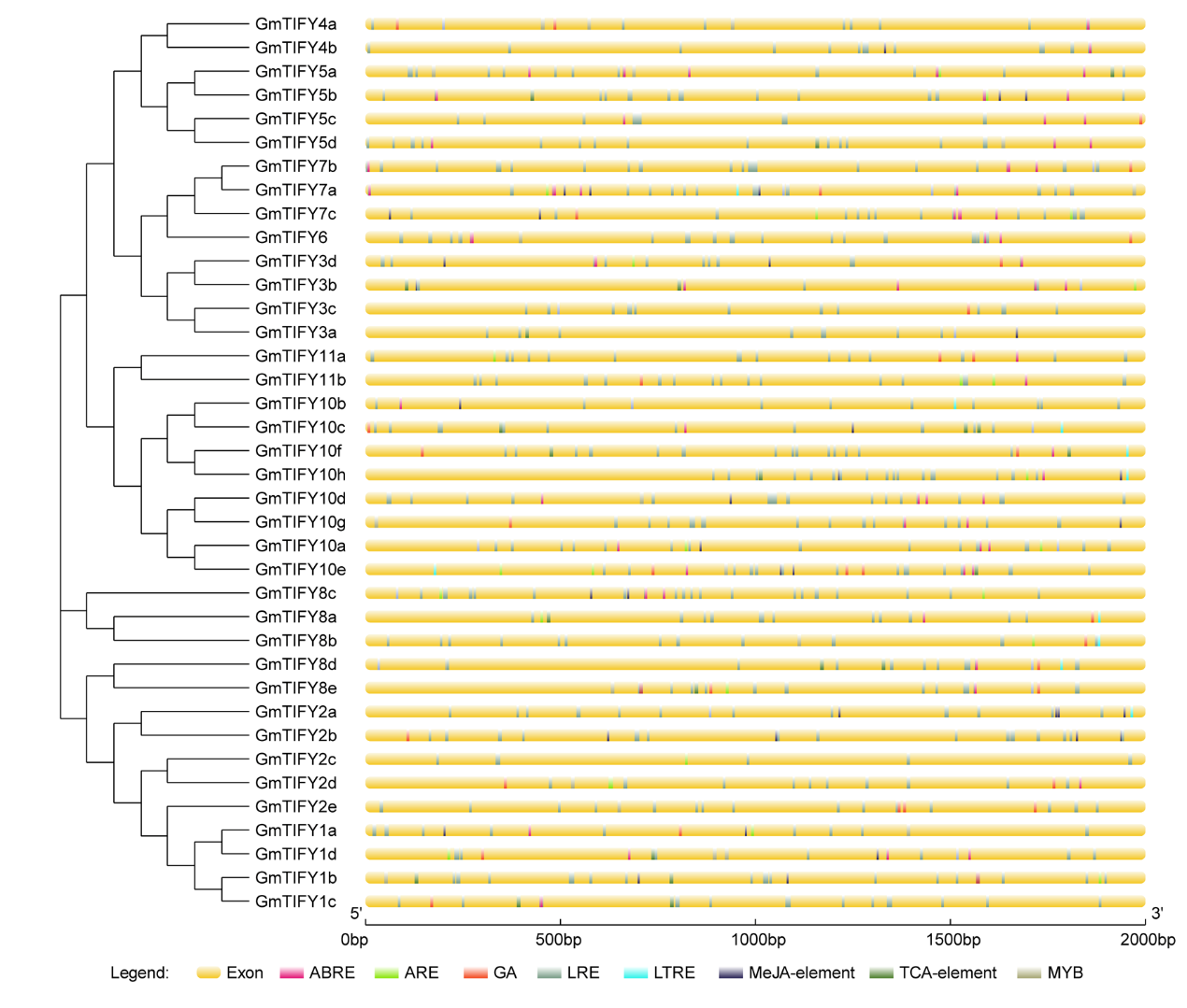
**Supplementary Figure 1.** Distribution of *cis*-acting elements in the promoters of 38 soybean *GmTIFY* genes. Promoters are indicated by yellow boxes and nine different-colored boxes represented nine different *cis*-acting elements. The scale at the bottom estimates the length of each protein.

**Supplementary Figure 2**


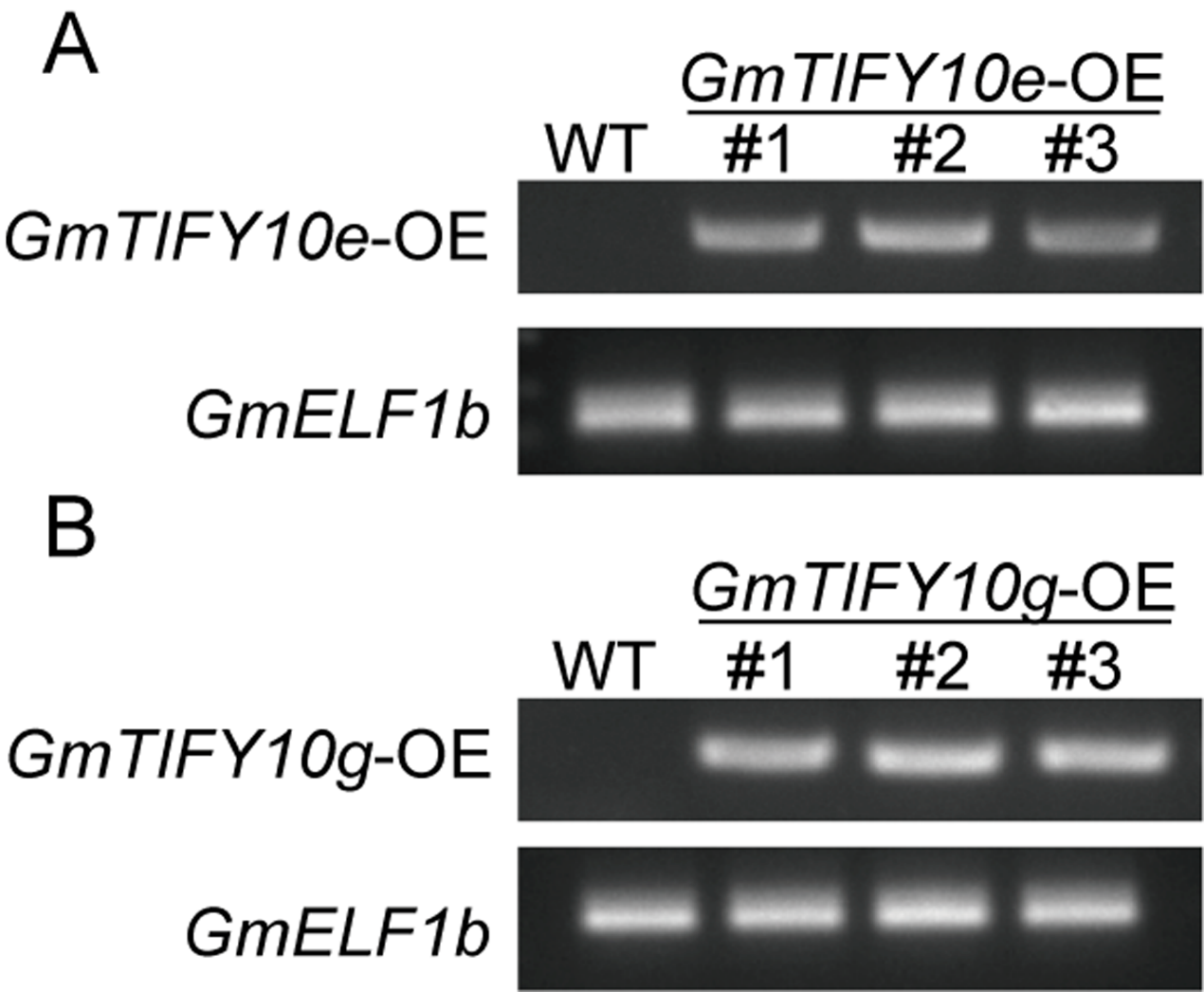


**Supplementary Figure 2.** Identification analysis of transgenic *Arabidopsis* lines. (A) Identification analysis of three *GmTIFY10e* transgenic *Arabidopsis* lines. (B) Identification analysis of three *GmTIFY10g* transgenic *Arabidopsis* lines. *GmELF1b* as the reference gene.

**Supplementary Figure 3**


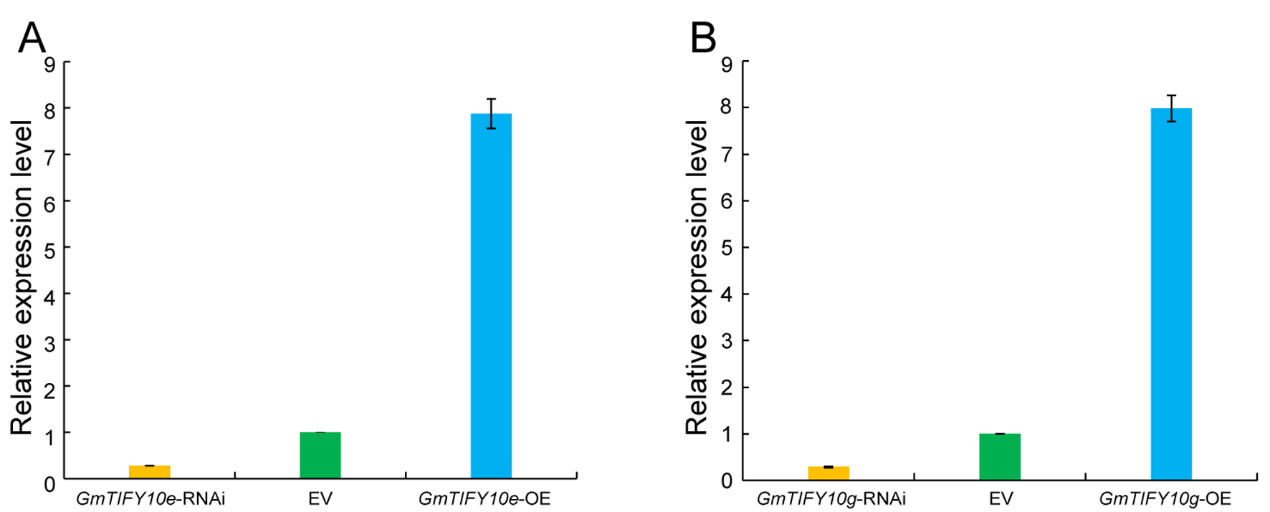


**Supplementary Figure 3.** RT-qPCR analysis of *GmTIFY10e* and *GmTIFY10g* expression levels in RNAi, EV and OE transgenic hairy roots. The data are shown as means of three biology repeats ± SD.

**Supplementary Figure 4**


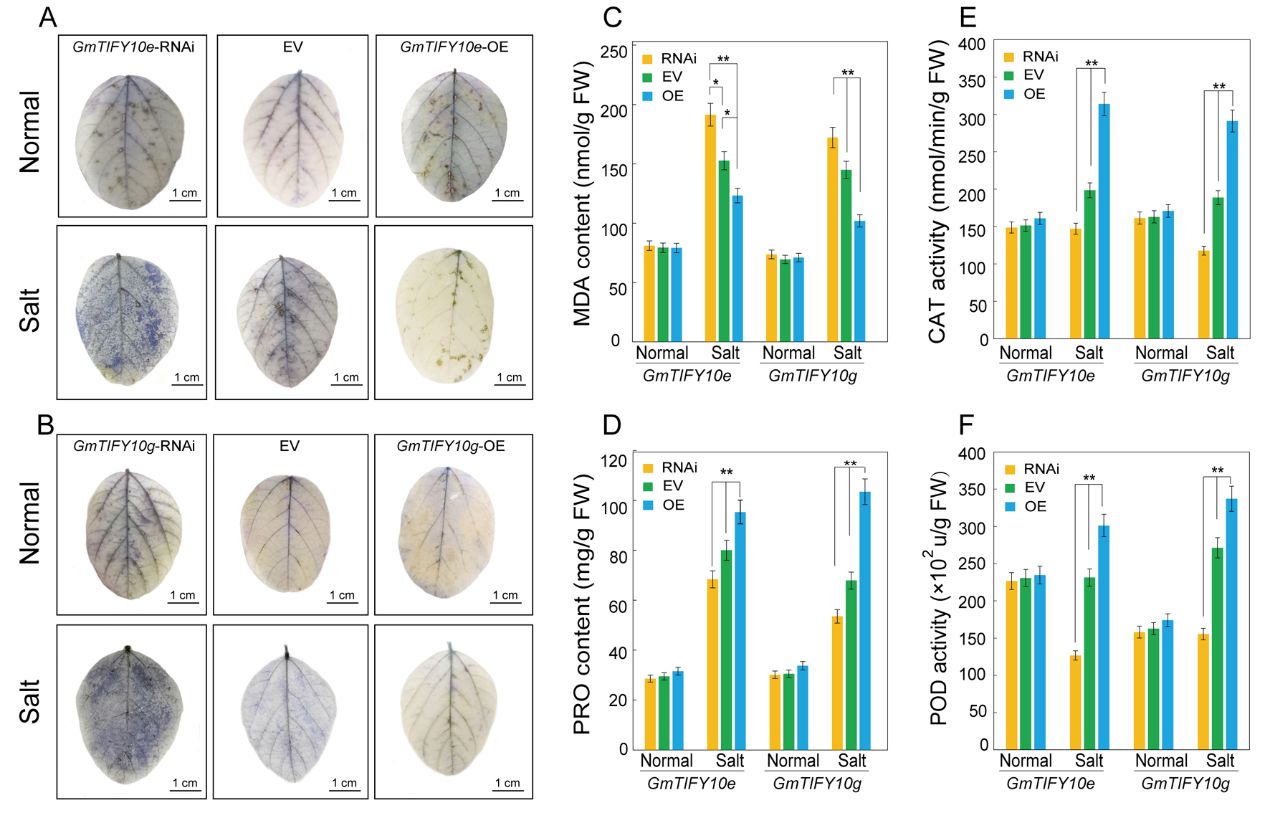
**Supplementary Figure 4.** Analysis of the function of soybean GmTIFY10e and GmTIFY10g under normal and salt stress. (A-B) NBT staining of EV and transgenic plant leaves under normal and salt stress. The scale bar indicates 1 cm. (C-F) The MDA (C), PRO (D), CAT (E) and POD (F) contents of EV and transgenic plant leaves under normal and salt stresses. The data are shown as the means ±SD obtained from three biological replicates. ANOVA test demonstrates that there are significant differences (*p<0.05, **p<0.01).

**Supplementary Figure 5**

**
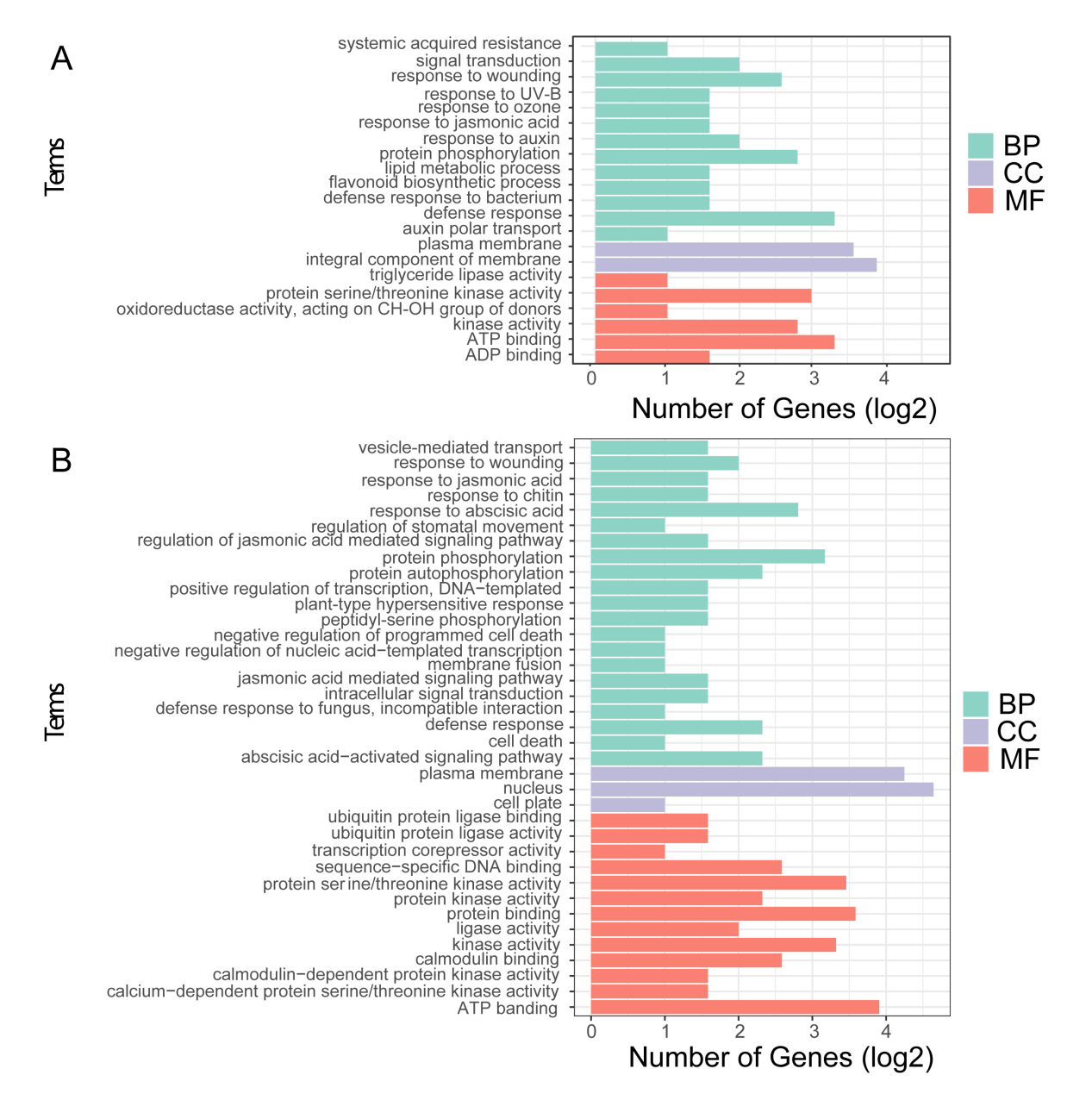
**

**Supplementary Figure 5.** Co-expression gene ontology (GO) enrichment analysis of *GmTIFY10e* and *GmTIFY10g* **(A)** GO enrichment analysis of *GmTIFY10e*. **(B)** GO enrichment analysis of *GmTIFY10g*. The BP, CC and MF indicate biological process, cellular component and molecular function, respectively.
